# An Asymptomatic Case of Radiologically Active Neurocysticercosis

**DOI:** 10.7759/cureus.8219

**Published:** 2020-05-21

**Authors:** Joshua A Ronen, Anusha Ammu, Gautham Gadiraju, Ajay Vaikuntam

**Affiliations:** 1 Internal Medicine, Texas Tech University Health Sciences Center at Permian Basin, Odessa, USA; 2 Internal Medicine, Midland Memorial Hospital, Midland, USA

**Keywords:** neurocysticercosis, ncc, praziquantal, albendazole, antihelminthics, hydrocephalus, dexamethasone, prednisone

## Abstract

The case describes an 82-year-old right-handed Hispanic male with multiple chronic comorbidities complaining of upper and lower extremity weakness as well as paresthesias that had been worsening over the last two weeks. He had bilateral upper and lower extremity weakness that was worse on the right and he complained of not being able to walk on his own with several falls preceding admission because of this. There were no overt signs of spinal cord compression or cauda equina syndrome. Laboratory work-up was unremarkable with exception to mildly elevated acute phase reactants. Computed tomography (CT) and magnetic resonance (MR) imaging later showed critical cervical compressive myelopathy at the C3-C4 level and multiple parenchymal and extra-axial enhancing lesions in the frontal lobes with associated calcifications suspicious for neurocysticercosis. Antihelminthic therapy was started and the patient underwent spinal fusion surgery with neurosurgery with an uncomplicated post-operative course - also lacking seizure activity of any kind.

## Introduction

Cysticercosis is a parasitic disease with intraparenchymal and extraparenchymal forms endemic to Asia, Central America, South America, and sub-Saharan Africa. It develops following the ingestion of eggs of the pork tapeworm, *Taenia solium* (*T. solium*). Tissue cystercerci develop at multiple sites in the body over a period of one to two months that can be at multiple stages of growth at a given time point from ingestion [1]. The phases of cysticercosis itself include viable, degenerating, and nonviable. The latter two stages are associated with the primary clinical manifestations of this disease, including increased intracranial pressures (ICP), hydrocephalus, and focal recurrent seizures [2]. 

The most common form of cysticercosis is the intraparenchymal type (neurocysticercosis, NCC), making up more than 60% of cases. In the United States, most patients with NCC present with a single enhancing lesion whereas multiple calcified lesions are found in said endemic areas [1]. Computed tomography (CT) and magnetic resonance (MR) imaging studies help establish the diagnosis along with serologies and the patient's presenting symptoms. It is important to also consider that negative serologies do not necessarily exclude the diagnosis as long as clinically significant examination and neuroimaging findings are present. Peripheral eosinophilia is generally absent and stool studies are insensitive [1].

Antihelminthic therapy is contraindicated should the patient display signs of elevated ICP such as altered sensorium, nausea, vomiting, or headache. High cyst burden, untreated hydrocephalus, and the presence of only calcified lesions are also absolute contraindications [2]. Should the patient present with obstructive hydrocephalus, neurosurgical intervention is the primary objective. Otherwise, the choice of pharmacotherapy consists of albendazole and corticosteroids for 10-14 days. Antiepileptic therapy is generally not recommended unless a patient presents with seizure activity.

## Case presentation

The patient is an 82-year-old Hispanic male with a past medical history of hyperlipidemia, hypertension, diabetes mellitus, and chronic kidney disease stage IV who presented to the emergency department complaining of bilateral upper extremity and lower extremity weakness and paresthesias for two weeks preceding admission. The patient had multiple falls recently after the onset of these symptoms. His symptoms had gotten to the point where he could not use his walker or even get up from a chair to walk. He denied headaches, diplopia, fevers, chest pain, shortness of breath, nausea, vomiting, dizziness, bowel incontinence, or bladder incontinence. He also denied any recent travel or consumption of undercooked meats. Vital signs were as follows: temperature of 36.8 °C, heart rate of 58 beats per minute sinus rhythm, respiratory rate of 14 breaths/min, blood pressure of 152/65, saturating 97% on room air. His physical examination was most significant for motor weakness of the bilateral upper and lower extremity, +3/5 on the right versus +4/5 on the left. Grip strength was tested in the upper extremity while dorsiflexion and plantarflexion were tested in the lower extremity. Pronator drift exam was negative.

**Figure 1:**
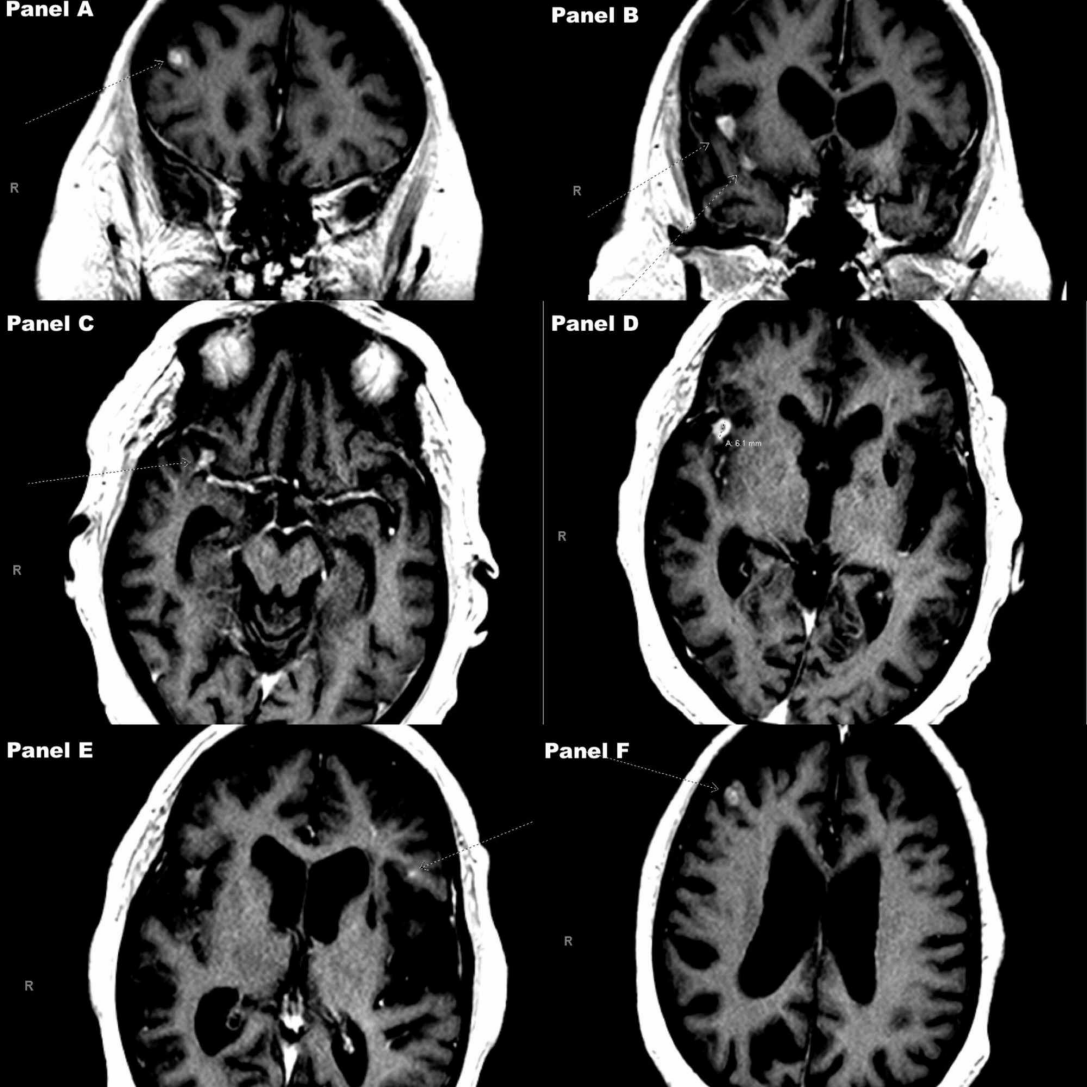
Diffusion weighted images (DWI) on MR of the brain with and without gadolinium contrast Magnify image to view arrows pointing out the intraparenchymal lesions themselves. Panels A and B show coronal cuts. Panels C through F show axial cuts.

His laboratory studies were not significant for any acute findings. Erythrocyte sedimentation rate (ESR) and C-reactive protein (CRP) were mildly elevated and a complete blood count with differential (CBC with diff) and coagulation profile were within normal limits (Table 1). Serology for cysticercosis IgG resulted as negative. CT scan of the head without contrast was negative for acute intracranial abnormalities. There was an old infarct detected in the left internal capsule region. Also noted were chronic right frontal cortical calcifications and anterior right temporal lobe calcifications that were thought to be related to an old infection or hemorrhage. CT angiogram of the head and neck revealed 60% stenosis at the left common carotid artery (CCA) origin and proximal left internal carotid artery (ICA). MR imaging of the cervical spine (c-spine) with and without contrast showed multilevel cervical spondylosis with severe C3-C4 and moderate to severe C6-C7 thecal sac stenosis. Subtle increased cord signals at C3-C4 were concerning for compressive myelopathy. A neurosurgical consultation was obtained and C3-C4 anterior spinal fusion with intervertebral cage placement was performed. MRI of the brain with and without contrast showed multiple subcentimeter parenchymal and extra-axial enhancing lesions in the frontal lobes and along the right sylvian fissure with associated calcifications concerning for active neurocysticercosis (Figure 1). The largest lesion along the right insula measured 7 mm with signal characteristics of a small abscess. An Infectious Disease consult was obtained and the patient was started on albendazole and praziquantal by mouth for ten days in total with dexamethasone. His hospital course was uncomplicated with no seizure activity reported. He worked well with physical therapy postoperatively and his bilateral upper and lower extremity weakness improved.

**Table 1:** Laboratory results Values described above are normal unless otherwise noted - (H): high; (L): low. Reference ranges for the following: ESR: 1 - 20 mm/h; CRP: 0 - 1.0 mg/dL; Cysticercosis Ab, IgG: see below - 0.8 IV or less: Negative - No significant level of cysticercosis IgG detected. - 0.9 - 1.1 IV: Equivocal - Questionable presence of cysticercosis IgG detected. - 1.2 IV or greater: Positive - IgG antibody to cysticercosis detected which may suggest current or past infection.

Test	Result
White Blood Cells (WBCs)	7.56 x 10^3^/uL
Hemoglobin	13.2 g/dL (L)
Hematocrit	39.4% (L)
Platelets	256 x 10^3^/uL
Eosinophils	1.6%
Erythrocyte Sedimentation Rate (ESR)	25 mm/h (H)
C-Reactive Protein (CRP)	3.0 mg/dL (H)
Prothrombin time (PT)	10.3 seconds
International Normalized Ratio (INR)	0.98 (L)
Glucose	117 mg/dL (H)
Blood Urea Nitrogen (BUN)	41 mg/dL (H)
Creatinine	1.3 mg/dL
Sodium	131 mEq/L (L)
Potassium	4.2 mEq/L
Chloride	94 mEq/L (L)
Bicarbonate	27 mEq/L
Glycated haemoglobin (HbA1c)	8.2% (H)
Cysticercosis Ab, immunoglobulin G (IgG) by enzyme-linked immunosorbent assay (ELISA)	0.2 IV

## Discussion

Per Gripper and Wilburn, cysticercosis is the most common parasitic disease of the human central nervous system (CNS) [3-4]. It is divided into parenchymal (neurocysticercosis, NCC) and extraparenchymal forms (intraventricular, subarachnoid, spinal, and ocular disease) and endemic to Asia, Central America, South America, and sub-Saharan Africa [3]. However, it is a commonly missed diagnosis in developed worlds. Cysticercosis develops following the ingestion of eggs of the pork tapeworm, *T. solium*. All in all, cases tend to be subclinical and arise in more rural communities with poor sanitation as well as those that have contact with free-roaming pigs [3-4]. There have been around 18,000 cases recorded in the United States between 2003-2012.

Tissue cystercerci develop at multiple sites in the body over a period of one to two months that can be at multiple stages of growth at a given time point from ingestion. The phases of cysticercosis itself include viable, degenerating, and nonviable [1]. The viable stage persists for many years before the cysticerci cannot evade the host immune response anymore in the intraparenchymal space and degenerate. Del Brutto et al. describe major neuroimaging criteria for NCC, including cystic lesions less than 20 mm in diameter without discernable scolex (the anterior end of the tapeworm where it attaches to the host), enhancing lesions, multilobulated cysts in basal subarachnoid cisterns, and typical parenchymal brain calcifications [5]. The presence of two different lesions helps strengthen the radiographic diagnosis. On neuroimaging, the viable cystercerci appear as 5-20 millimeter (mm) hypodense lesions on CT scans [2]. These do not enhance after contrast administration and do not cause much inflammation in the surrounding tissues. The host inflammatory response is one of two triggers for seizure onset when the degeneration phase takes place. The presence of the inflammatory response goes hand-in-hand with contrast enhancement on CT. The second of the two triggers can take place during the nonviable phase of this disease. This phase is characterized by calcified granuloma formation. These granulomas are most often 2-4 mm in diameter and are nonenhancing [2]. They can be associated with some perilesional edema. The findings on our patient’s CT scan of the brain without contrast included chronic right frontal and right anterior temporal calcifications suggestive of an “old infection.” The findings on his MRI of the brain with and without contrast suggested multiple subcentimeter parenchymal enhancing lesions and others with associated calcifications. In the United States, most patients with NCC present with a single enhancing lesion whereas multiple calcified lesions are found in the endemic areas previously described. Based on neuroimaging, our patient had multiple lesions in the degenerating and nonviable phases of their life cycle both of which can serve as triggers for seizures in the setting of this illness, but he remained free of them.

The most common form of cysticercosis is the intraparenchymal type, making up more than 60% of cases [1]. Ultimately, the neurologic manifestations of NCC are dependent on where the cortical lesions are present and the degree of the inflammatory response [6]. Rodriguez-Hidalgo et al. point out that the typical incubation period for intestinal *T. solium* is 83 days alone [7]. Between the primary date of ingestion of the tapeworm and when symptoms arise, it could be between three to as many as 30 or more years [1]. Notably, there are more calcified lesions in patients like ours with delayed presentations. Seizures that are seen in context of this disease are focal in nature. They are the most common clinical manifestation. The diagnosis of NCC is made with signs of increased ICP, physical exam findings (ie. seizures), corroborating objective data collected at the time of admission (CT and MR neuroimaging), and epidemiologic proof of exposure. Serologies (more specifically enzyme-linked immunotransfer blots (EITBs)) have 98% sensitivity and 100% specificity to confirm the diagnosis when imaging results are consistent but not diagnostic of NCC and when there is more then one live cyst or subarachnoid disease [1,6]. The EITB tests for antibodies to seven specific larval antigens, per Rajshekar [6]. It is important to also consider that negative serologies (such as in this case) do not necessarily exclude the diagnosis as long as clinically significant examination and neuroimaging findings are present. Peripheral eosinophilia is generally absent and stool studies are insensitive [1]. 

Management

Once the diagnosis is confirmed, patients should have an ophthalmologic exam to exclude ocular disease which can threaten vision when antihelminthic therapy ensues. Those patients who are anticipated to need long term corticosteroid therapy should also be tested for latent turberculosis infection (LTBI) and Strongyloides infection [2]. As far as medical therapy is concerned, it should be catered to each unique clinical scenario. If the patient displays signs of elevated ICP such as altered sensorium, nausea, vomiting, or headache - antihelminthic therapy is contraindicated. High cyst burden, untreated hydrocephalus, and presence of only calcified lesions are also absolute contraindications [2]. This is noteworthy as patients with parenchymal disease (NCC) can present with diffuse cerebral edema that necessitates supplementation of dexamethasone at a dose of 0.2 to 0.4 mg/kg/day. Once the inflammation resolves, initiation of antihelminthic therapy can be re-considered. In the absence of seizure activity, anti-epileptic medications need not be started. Viable cystercerci can produce a state of obstructive hydrocephalus which necessitates surgical intervention [2]. While antihelminthic treatment does increase risk of recurrent focal seizure activity (moreso in patients with multiple parenchymal lesions), in the long term this treatment reduces this risk as well as the burden of active lesions and recurrence of hydrocephalus. The choice of antihelminthic regimen depends on the cyst burden and the duration of treatment is 10-14 days. Per the American Academy of Neurology as Rizvi et al. outline - with 1-2 viable or degenerating cysts, albendazole 15 mg/kg per day split into two daily doses (max: 1.2 g/day) is prescribed. In patients with more than two cysts, the same prescription for albendazole is used with praziquantal 50 mg/kg split into three daily doses [2,8]. While neurological exacerbations (i,e. seizures) secondary to cysticidal therapy can occur at anytime, most have been described between days two through five of therapy [8]. Herein lies the reasoning for concurrent corticosteroid therapy with dexamethasone (0.1 mg/kg/day) or prednisone (1 mg/kg/day). Patients should receive their first dose of corticosteroids ideally one day prior to the initiation of antihelminthic therapy. They should then be tapered off rapidly once the antimicrobial treatment period concludes. Overall, Gripper and Wilburn explain that the parenchymal variant of this disease has a better prognosis than its extra-parenchymal counterpart [4]. Patients should be screened for persistence of larval antigens using the antigen-enzyme linked immunosorbent assay (Ag-ELISA) test within two months of completing treatment [7].

## Conclusions

Present estimations of the number of neurocysticercosis cases worldwide are insufficient to quantify the actual disease burden. While its incidence is not high in developed countries such as the United States, its management bears great attention as certain presentations can be immediately life-threatening. There is no shortage in supply of anti-helminthic therapies such as praziquantel and albendazole. Nevertheless, clinicians must still have a lower threshold to image patients with suggestive presentations, prompting early neurosurgical evaluation and intervention if indicated. Asymptomatic cases of incidentally found degenerating or nonviable CNS disease should still garner Infectious Disease consultation. Patients from endemic areas will seek medical attention in urban centers with established healthcare systems. From a public health standpoint, it would be helpful to track such patients as far as their whereabouts and contacts prior to presentation. This way, officials at the city and state level will be able to employ population health strategies to reduce the presence of risk factors that subject certain groups of individuals to exposure to the pork tapeworm and subsequent morbidity.
